# Stacked Autoencoders for the P300 Component Detection

**DOI:** 10.3389/fnins.2017.00302

**Published:** 2017-05-30

**Authors:** Lukáš Vařeka, Pavel Mautner

**Affiliations:** Neuroinformatics Research Group, Department of Computer Science and Engineering, Faculty of Applied Sciences, University of West BohemiaPilsen, Czechia

**Keywords:** brain-computer interfaces, deep learning, event-related potentials, machine learning, P300, stacked autoencoders

## Abstract

Novel neural network training methods (commonly referred to as deep learning) have emerged in recent years. Using a combination of unsupervised pre-training and subsequent fine-tuning, deep neural networks have become one of the most reliable classification methods. Since deep neural networks are especially powerful for high-dimensional and non-linear feature vectors, electroencephalography (EEG) and event-related potentials (ERPs) are one of the promising applications. Furthermore, to the authors' best knowledge, there are very few papers that study deep neural networks for EEG/ERP data. The aim of the experiments subsequently presented was to verify if deep learning-based models can also perform well for single trial P300 classification with possible application to P300-based brain-computer interfaces. The P300 data used were recorded in the EEG/ERP laboratory at the Department of Computer Science and Engineering, University of West Bohemia, and are publicly available. Stacked autoencoders (SAEs) were implemented and compared with some of the currently most reliable state-of-the-art methods, such as LDA and multi-layer perceptron (MLP). The parameters of stacked autoencoders were optimized empirically. The layers were inserted one by one and at the end, the last layer was replaced by a supervised softmax classifier. Subsequently, fine-tuning using backpropagation was performed. The architecture of the neural network was 209-130-100-50-20-2. The classifiers were trained on a dataset merged from four subjects and subsequently tested on different 11 subjects without further training. The trained SAE achieved 69.2% accuracy that was higher (*p* < 0.01) than the accuracy of MLP (64.9%) and LDA (65.9%). The recall of 58.8% was slightly higher when compared with MLP (56.2%) and LDA (58.4%). Therefore, SAEs could be preferable to other state-of-the-art classifiers for high-dimensional event-related potential feature vectors.

## 1. Introduction

**Figure 1:**
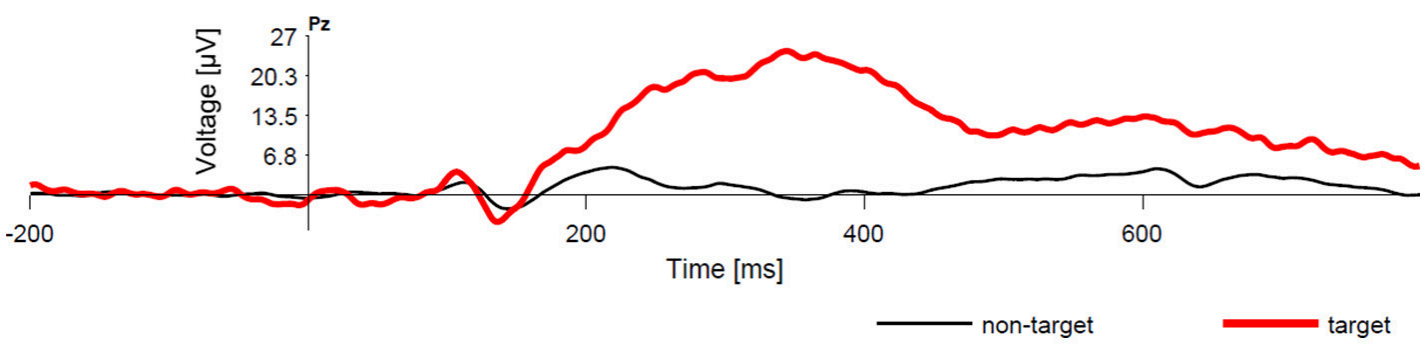
Comparison of averaged EEG responses to common (non-target) stimuli and rare (target) stimuli. There is a clear P300 component following the target stimuli.

A brain-computer interface (BCI) enables communication without movement based on brain signals measured with electroencephalography (EEG). One of the most widespread BCI paradigms relies on the P300 event-related potentials, and is referred to as P300 BCIs. The P300 is an event-related potential elicited by oddball paradigm (see Figure 1). It exhibits larger amplitudes in target (rare) stimuli (Fazel-Rezai et al., 2012). Because the P300 component can also be observed for stimuli that are selected by the user e.g., because of his/her intention, many different BCIs can be designed based on this principle. The P300 speller introduced by Farwell and Donchin (1988) can serve as one of the examples. Furthermore, P300 BCIs have consistently exhibited several useful features—they are relatively fast, straightforward, and require practically no training of the user (Fazel-Rezai et al., 2012). Unfortunately, the detection of the P300 is challenging because the P300 component is usually hidden in underlying EEG signal (Luck, 2005). Therefore, well-trained machine learning system is one of the most important parts of any P300 BCI system. Its task is to read EEG patterns and discriminate them into two classification classes (i.e., P300 detected, P300 not detected).

**Figure 2:**
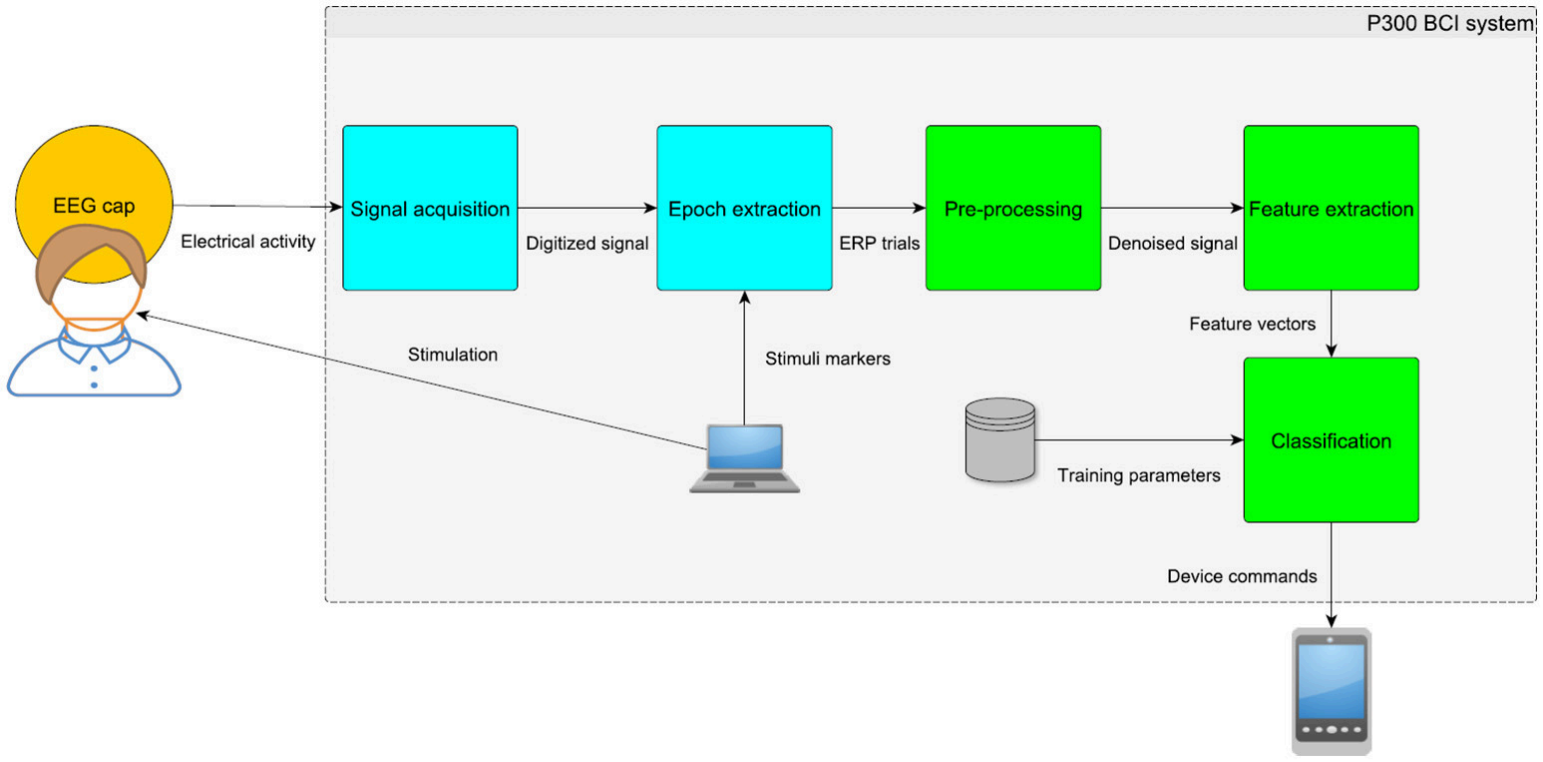
Diagram of the P300 BCI system. The EEG signal is captured, amplified and digitized using equidistant time intervals. Then, the parts of the signal time-locked to stimuli (i.e., epochs or ERP trials) must be extracted. Preprocessing and feature extraction methods are applied to the resulting ERP trials in order to extract relevant features. Classification uses learned parameters (e.g., distribution of different classes in the training set) to translate the feature vectors into commands for different device types.

Typically, the P300 detection requires preprocessing, feature extraction, and classification (depicted in more detail in Figure 2). The objective of preprocessing is to increase signal to noise ratio. Bandpass filtering of raw EEG signals is a common preprocessing method in P300 detection systems. Since the P300 component is stimulus-locked and the background activity is randomly distributed, the P300 waveform can be extracted using averaging (Luck, 2005). Averaging gradually improves signal to noise ratio. On the other hand, averaging also slows down the bit-rate of P300 BCI systems and distorts the shape of ERPs (Luck, 2005). Then, features are extracted from EEG signals. Different methods have been used for this purpose, e.g., discrete wavelet transform, independent component analysis, or principal component analysis. The final step is classification. Farwell and Donchin used step-wise discriminant analysis (SWDA) followed by peak picking and covariance evaluation (Farwell and Donchin, 1988). Other methods have also been used for the P300 detection such as support vector machine (SVM) (Thulasidas et al., 2006), and linear discriminant analysis (LDA) (Guger et al., 2009). Although different features and classifiers have been compared (Mirghasemi et al., 2006), comparisons of all different features extraction and classification methods applied to the same data set have only been published rarely. One study has, however, examined this issue. In Krusienski et al. (2006) it was shown that SWDA and Fisher's linear discriminant (FLD) provided the best overall performance and implementation characteristics for practical classification, as compared to Pearson's correlation method (PCM), a linear support vector machine (LSVM), and a Gaussian kernel support vector machine (GSVM) (Fazel-Rezai et al., 2012). In Manyakov et al. (2011), the authors demonstrated that LDA and Bayesian linear discriminant analysis (BLDA) were able to beat other classification algorithms. For comparison purposes, there is a benchmark P300 speller dataset from the BCI Competition 2003 (Blankertz et al., 2004) and some papers report results achieved on this dataset. Several approaches were able to reach 100% accuracy using only 4–8 averaged trials on the BCI Competition 2003 data (Cashero, 2012). In single trial P300 detection (i.e., detection without averaging the trials), the performance reported in the literature is lower—typically between 65 and 70% (Jansen et al., 2004; Haghighatpanah et al., 2013).

Recent development in the field of deep learning neural networks has opened new research possibilities regarding P300 BCI systems. Using a combination of unsupervised pre-training and subsequent fine-tuning, deep neural networks have become one of the most reliable classification methods, in some pattern recognition cases even outperforming other state-of-the-art methods (Pound et al., 2016). P300 feature vectors reflect the nature of EEG signal. They are high-dimensional, not linearly separable, consisting of both time samples and spatial information (by concatenating multiple EEG channels). Therefore, deep learning models seem appealing since they are especially powerful for high-dimensional and complex feature vectors. Furthermore, to the authors' best knowledge, there are very few papers that study deep neural networks for EEG/ERP data (Deng and Yu, 2013).

The objective of this paper is to verify if one of the deep learning models suitable for real-valued inputs—stacked autoencoders—is suitable for the detection of the P300 component, and to compare it with traditional classification approaches. The datasets used were previously freely provided to public. The paper is organized as follows: Section 2 introduces deep learning models including stacked autoencoders. Then the proposed experiment is described: in Section 2.3, details about the obtained data and experimental conditions are described. Section 2.4.1 explains feature extraction and Section 2.4.2 describes the procedure used to train stacked autoencoders and classification models that were used for comparison. Results are presented in Section 3 and discussed in Section 4.

## 2. Materials and methods

### 2.1. Deep learning

The main goal of this paper is to evaluate the benefits of using new deep learning models for P300 BCIs. Therefore, in this section, deep learning models are introduced.

Deep learning models have emerged as a new area of machine learning since 2006 (Deng and Yu, 2013). For complex and non-linear problems, deep learning models have proven to outperform traditional classification approaches (e.g., SVM) that are affected by the curse of dimensionality (Arnold et al., 2011). These problems cannot be efficiently solved by using neural networks with many layers (commonly referred to as deep neural networks) trained using backpropagation. The more layers the neural network contains, the lesser the impact of the backpropagation on the first layers. The gradient descent then tends to get stuck in local minima or plateaus which is why no more than two layer were used in most practical applications (Deng and Yu, 2013).

In deep learning, each layer is treated separately and successively trained in a greedy way: once the previous layers have been trained, a new layer is trained from the encoding of the input data by the previous layers. Then, a supervised fine-tuning stage of the whole network can be performed (Arnold et al., 2011). Deep networks models generally fall into the following categories (Arnold et al., 2011):

- Deep belief networks (stacked restricted Boltzmann machine)
- Stacked autoencoders
- Deep Kernel Machines
- Deep Convolutional Networks

The main goal of this paper is to explore stacked autoencoders for this task. Deep belief networks from deep learning category have already been successfully applied to P300 classification (Sobhani, 2014). However, to the authors best knowledge, stacked autoencoders have so far not been used for the P300 detection. Furthermore, they use real inputs which is suitable for this application.

### 2.2. Stacked autoencoders

**Figure 3:**
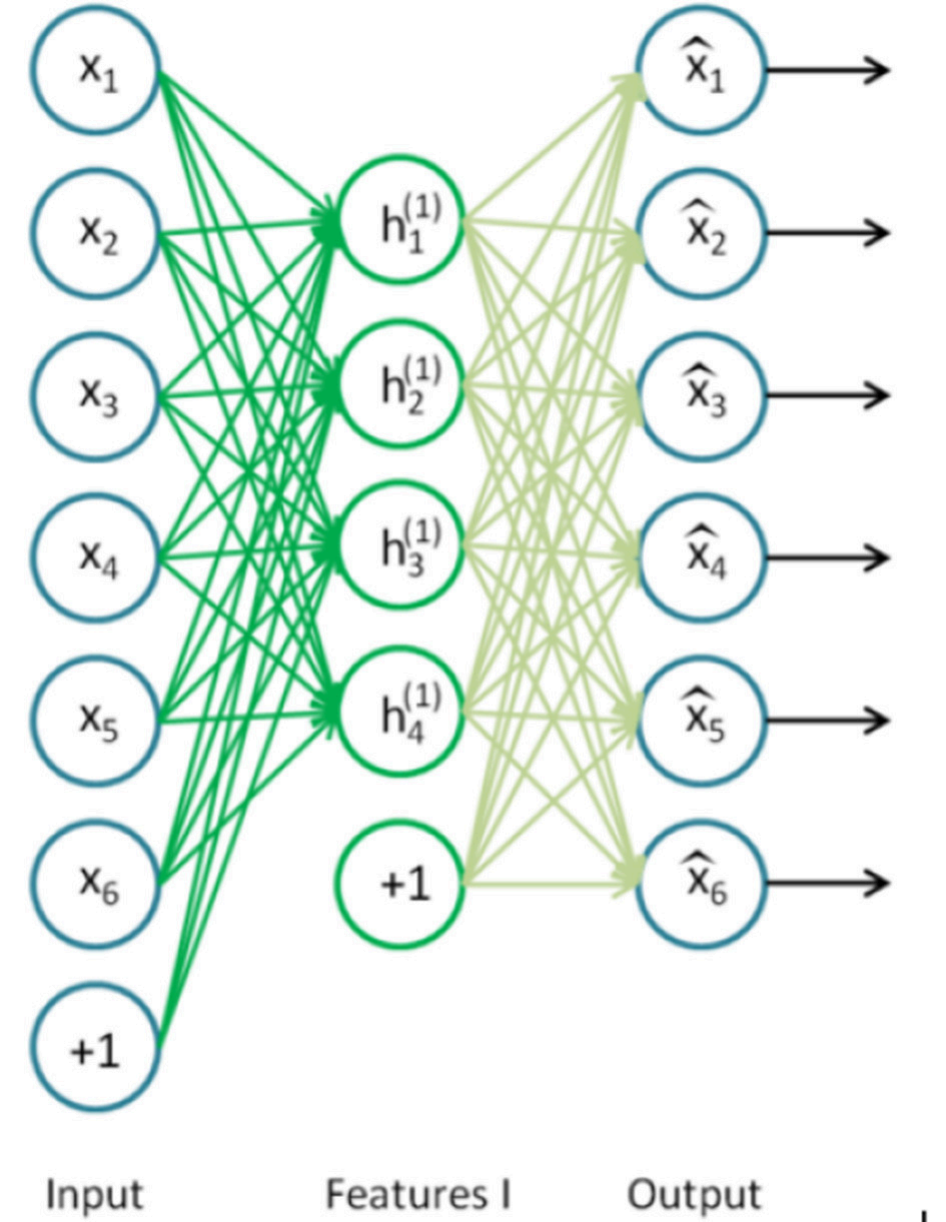
Autoencoder. The input layer (*x*_1_, *x*_2_, .. , *x*_6_) has the same dimensionality as the output (decoding layer). The encoding layer ($h_{1}^{\left(1\right)}$, .., $h_{4}^{\left(1\right)}$) has a lower dimensionality and performs the encoding (Ng et al., 2010).

A single autoencoder (AA) is a two-layer neural network (see Figure 3). The encoding layer encodes the inputs of the network and the decoding layer decodes (reconstructs) the inputs. Consequently, the number of neurons in the decoding layer is equal to the input dimensionality. The goal of an AA is to compute a code *h* of an input instance *x* from which *x* can be recovered with high accuracy. This models a two-stage approximation to the identity function (Arnold et al., 2011):

(1) $$\begin{array}{r} f_{dec}\left(f_{enc}\left(x\right)\right)=f_{dec}\left(h\right)=\hat{x}\approx x \end{array}$$

with *f*_*enc*_ being the function computed by the encoding layer and *f*_*dec*_ being the function computed by the decoding layer.

The number of neurons in the encoding layer is lower than the input dimensionality. Therefore, in this layer, the network is forced to remove redundancy from the input by reducing dimensionality. The single autoencoder (being a shallow neural network) can easily be trained using standard backpropagation algorithm with random weight initialization (Ng et al., 2010).

Stacking of autoencoders in order to boost performance of deep networks was originally proposed in Bengio et al. (2007).

**Figure 4:**
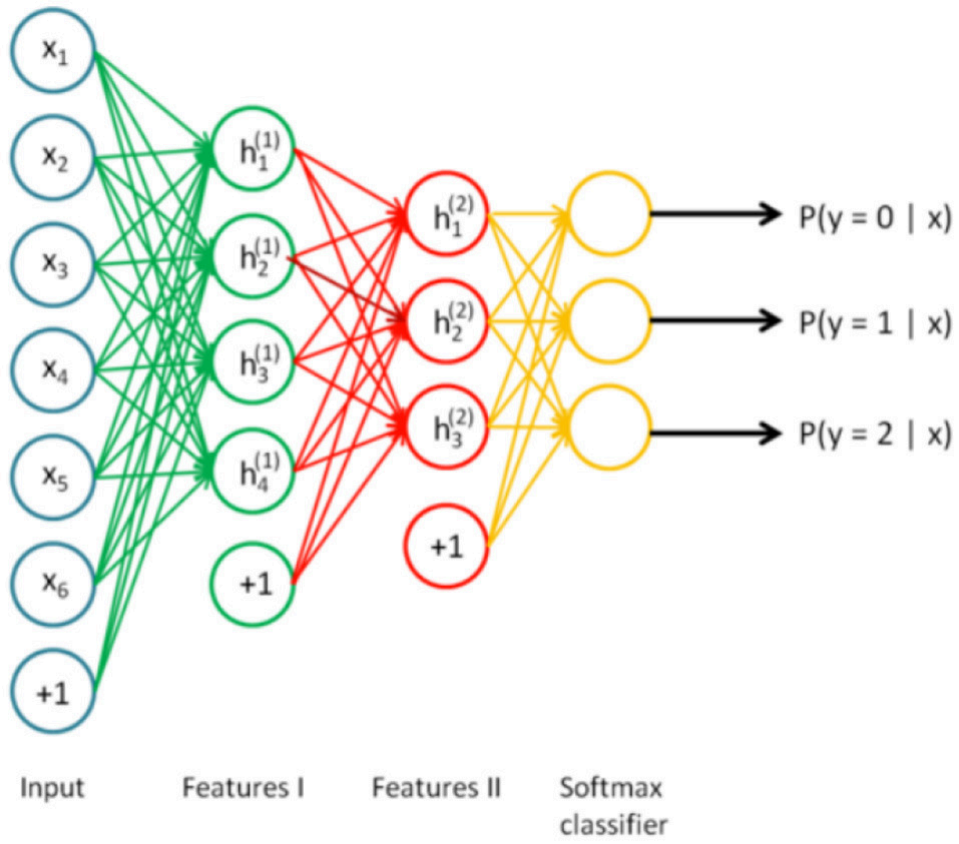
**Stacked autoencoder (Ng et al., 2010)**.

A key function of stacked autoencoders is unsupervised pre-training, layer by layer, as input is fed through. Once the first layer is pre-trained (neurons $h_{1}^{\left(1\right)}$, $h_{2}^{\left(1\right)}$, .., $h_{4}^{\left(1\right)}$ in Figure 3), it can be used as an input of the next autoencoder. The final layer can deal with traditional supervised classification and the pretrained neural network can be fine-tuned using backpropagation. Stacked autoencoder is depicted in Figure 4 (Ng et al., 2010).

### 2.3. Experimental design

#### 2.3.1. Introduction

To compare stacked autoencoders with traditional classification models, an ERP experiment was designed and conducted in our laboratory to obtain P300 data for training and testing of the classifiers used. The data with corresponding metadata and detailed description are available in Vareka et al. (2014a).

#### 2.3.2. Stimulation device

The stimulation device (Dudacek et al., 2011) was designed at our department. The main part of the stimulation device is a box containing three high-power Light-Emitting Diodes (LEDs) differing in their color: red, green, and yellow.

The core of the stimulator is an 8bit micro-controller that generates the required stimuli. The control panel consists of a LCD display and a set of push-buttons that are used to set the parameters of the stimulation protocol. The stimulator also generates additional synchronization signals for the EEG recorder.

**Figure 5:**
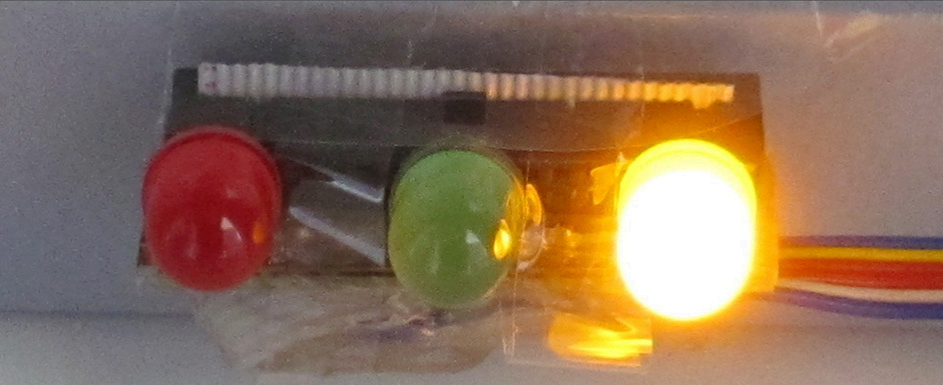
Stimulation device with flashing diodes.

The stimulator has typically been used for modified odd-ball paradigm experiments (three stimulus paradigm Dudacek et al., 2011). Apart from traditional target and non-target stimuli, the device can also randomly insert distractor stimuli. The distractor stimuli are usually used to elicit the subcomponent of the P3 waveform (called P3a) (Polich, 2007). Figure 5 shows the LED module with the yellow diode flashing.

#### 2.3.3. Stimulation protocol

The stimulation protocol uses the device described above. For our experiments, the following setting of the stimulation device was used: each diode flashes once a second and each flash takes 500 ms. The probabilities of the red, green and yellow diodes flashing were 83, 13.5, and 3.5%, respectively. Consequently, the green diode was the target stimulus and the red diode the non-target stimulus. The yellow diode was the distractor stimulus, and was ignored in the subsequent processing. The participants were sitting 1 m from the stimulation device for 20 min. The experimental protocol was divided into three phases, each containing 30 target stimuli and each about 5 min long. There was a short break between each two phases. The participants were asked to sit comfortably, not to move and to limit their eye blinking. They were instructed to pay attention to the stimulation device and not to perform another task-relevant cognitive or behavioral activity.

#### 2.3.4. Procedure

The following experimental procedure was applied: Each participant was acquainted with the course of the experiment and answered questions concerning his/her health. Then he or she was given the standard EEG cap made by the Electro-Cap International. The international 10–20 system of electrode placement was used. The participant was subsequently taken to the soundproof and electrically shielded cabin. The reference electrode was located at the root of his/her nose. The participant was told to watch the stimulator.

#### 2.3.5. Recording of the data

The BrainVision amplifier and related software for recording were used in the subsequent experiments. The data were obtained with the following parameters: the sampling rate was set to 1 kHz, the number of channels was set to 19, the resolution was set to 0.1 μV and the recording low-pass filter was set with the cut-off frequency of 250 Hz. The impedance threshold was set to 10 *kΩ*.

#### 2.3.6. Measured subjects

A group of 25 healthy individuals (university students, aged 20–26) participated in our experiments. However, only the data from 15 subjects were used in subsequent experiments. Five subjects were rejected even before storing the data, so they are unavailable in our data publication (Vareka et al., 2014b). Those subjects were blinking excessively, inattentive and in some cases, the experiment was ended early. High impedance was also one of the reason for rejection, because it was typically associated with a complete data loss on one or more electrode. Other five subjects were rejected based on their lack of the P300 response. All subjects signed an agreement with the conditions of the experiment and with the sharing of their EEG/ERP data.

#### 2.3.7. Availability of the measured data

The experimental protocol and datasets supporting the results of this article are described in more detail in Vareka et al. (2014a). The datasets are available for download in the EEG/ERP Portal under the following “http://eegdatabase.kiv.zcu.cz/ (Moucek and Jezek, 2009). Supporting material for this paper can also be found in the GigaScience database, GigaDB (Vareka et al., 2014b).

### 2.4. Pattern recognition

#### 2.4.1. Preprocessing and feature extraction

For feature extraction, the Windowed means paradigm (Blankertz et al., 2011) was used. It is a modern method that includes features from multiple channels and the most significant time intervals. Its use for P300 BCIs was encouraged in Blankertz et al. (2011). The method is based on selecting epoch time windows that contain the components of interest (e.g., the P300 component). The following steps were taken:

1. Each dataset was split into epochs (trials) using stimuli markers of target events—the green diodes flashing (S 2) and non-target events—the red diodes flashing (S 4). Each trial started 500 ms before the stimulus, and ended 1,000 ms after the stimulus.
2. Baseline correction was performed by subtracting the average of 500 ms before the stimulus onset from each trial.
3. For averaging, 50 ms long time windows between 150 ms and 700 ms after the stimuli onset were selected. The intervals used were based on expected locations of the P300 and other cognitive ERP components (Luck, 2005) and further adjusted experimentally. Subsequently, 11 averages were extracted from all available 19 EEG channels.
4. Averages from all 19 channels were concatenated. As a result, each feature vector had dimensionality of 209.
5. Finally, each individual feature vector was normalized using its length.

The procedure for finding suitable parameters for the P300 detection based on the Windowed means paradigm is described in detail in Vareka and Mautner (2015). For example, it was investigated how to choose time intervals for averaging to maximize classification performance.

#### 2.4.2. Classification

For classification, the state-of-the-art methods for P300 BCIs mentioned e.g., in Lotte et al. (2007): linear discriminant analysis (LDA) and multi-layer perceptron (MLP) were compared with stacked autoencoders (SAEs).

The features vectors were extracted as described in Section 2.4.1. The training set was concatenated using the data from four subjects (experimental IDs 99, 100, 104, and 105, none of them included in the testing dataset). The datasets used for training were selected manually to contain an observable P300 component with different amplitudes and latencies. From each subject, all target trials were used. The corresponding number of non-targets was randomly selected from each subject. Consequently, the training dataset contained 366 target and 366 non-target trials. Finally, all training trials were randomly shuffled. Only the training set was used for both unsupervised pre-training and supervised fine-tuning. There were no further weight updates in the testing mode. Therefore, it could be observed if once trained classifiers can generalize for other subjects.

To optimize parameters for classification models, 20% randomly selected subset of the training dataset was used for validation. Then, manually selected parameters were inserted and the process of training and evaluating the results was repeated ten times to average the performance for each configuration. After the parameters were found, the models were trained on the whole training dataset and subsequently tested.

Matlab Neural Network Toolbox was used for the implementation of stacked autoencoders (MATLAB, 2015). The parameters of the stacked autoencoder (number of layers, number of neurons in each layer, and number of iterations for the hidden layers) were empirically optimized using the results on the validation set. The experimentation started with two layers, then either new neurons were added into the layer, or a new layer was added until the performance of the classifier stopped increasing.

Finally, the following procedure was used to train the network. The maximum number of training epochs was limited to 200.

1. The first autoencoder with 130 hidden neurons was trained.
2. The second autoencoder with 100 hidden neurons was connected with the first autoencoder to form a 209-130-100-209 neural network, and trained.
3. The third autoencoder with 50 hidden neurons was connected with the second autoencoder to form a 209-130-100-50-209 neural network, and trained.
4. The fourth autoencoder with 20 hidden neurons was connected with third autoencoder to form a 209-130-100-50-20-209 neural network, and trained.

Furthermore, the following parameters were set for the network globally to reduce overfitting and adjust the weight update: L2WeightRegularization was set to 0.004, SparsityRegularization was set to 4, and SparsityProportion was set to 0.2. These values were set according to common recommendations (MATLAB, 2015) and then slightly adjusted when tuning up the training.

**Figure 6:**
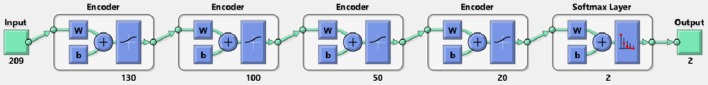
The structure of the SAE neural network.

After the training of each autoencoder, the input feature vectors were encoded using that autoencoder to form input vectors of the next autoencoder. Using the output of the last autoencoder, softmax supervised classifier was trained with 200 training iterations. Finally, the whole pre-trained 209-130-100-50-20-2 network was fine-tuned using backpropagation. The structure of the stacked autoencoder is depicted in Figure 6.

The same numbers of neurons for each layer were used for MLP. However, the phase of unsupervised pre-training was not included. Instead, the randomly initialized network was trained using backpropagation. The number of training iterations was empirically set to 1,000. For the training of LDA, the shrinkage regularization was used as recommended by Blankertz et al. (2011) to reduce the impact of the curse of dimensionality.

## 3. Results

To evaluate the results of classification, accuracy, precision and recall were calculated. Suppose that we have *t*_*p*_ - number of true positive detections, *t*_*n*_ - number of true negative detections, *f*_*p*_ - number of false positive detections, and *f*_*n*_ - number of false negative detections. The following values were calculated:

(2) $$\begin{array}{r} ACCURACY=\frac{t_{p}+t_{n}}{t_{p}+t_{n}+f_{p}+f_{n}} \end{array}$$

(3) $$\begin{array}{r} PRECISION=\frac{t_{p}}{t_{p}+f_{p}} \end{array}$$

(4) $$\begin{array}{r} RECALL=\frac{t_{p}}{t_{p}+f_{n}} \end{array}$$

**Figure 7:**
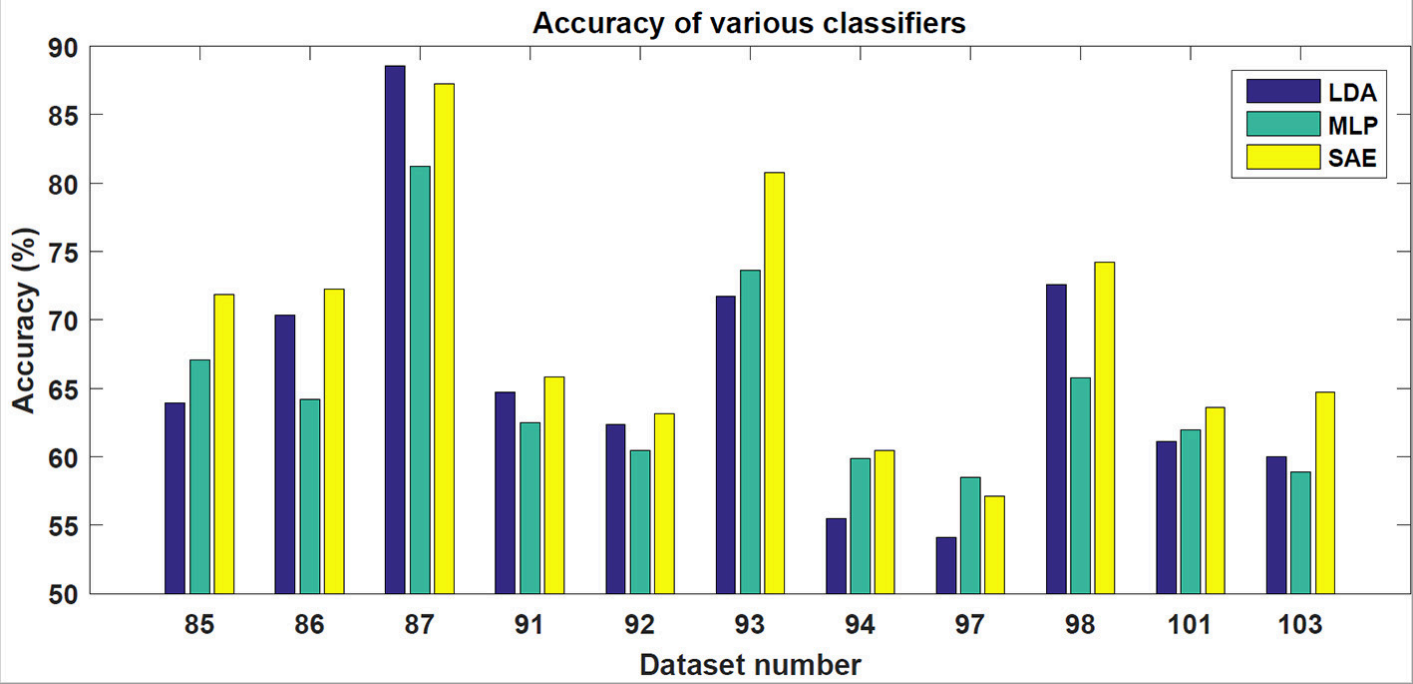
For each dataset from the testing set, the achieved accuracy for LDA, MLP, and SAE classifiers is depicted.

**Figure 8:**
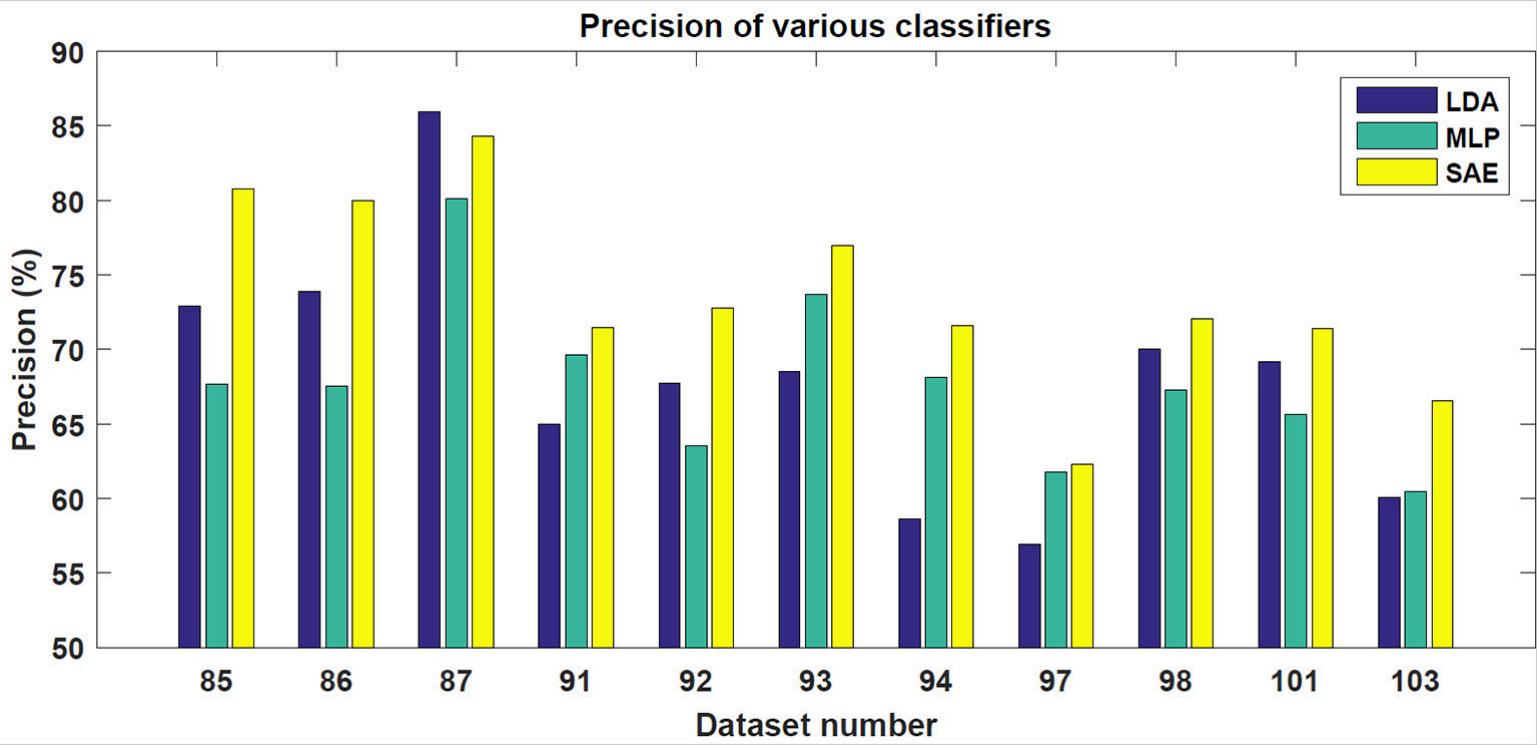
For each dataset from the testing set, the achieved precision for LDA, MLP, and SAE classifiers is depicted.

**Figure 9:**
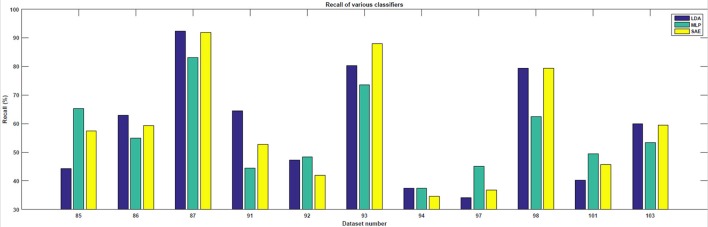
For each dataset from the testing set, the achieved recall for LDA, MLP, and SAE classifiers is depicted.

In the testing phase, the data from each experiment were evaluated. Similarly to the training dataset, all target trials were included but only the corresponding number of first non-target trials were used. The number of trials varied slightly for each subject. However, for each subject, ~90 target and 90 non-target trials were extracted. The results achieved are shown in Table 1. For each classifier, average accuracy, precision, and recall are listed. Figures 7–9 depict achieved classification accuracy, precision, and recall for each testing dataset, respectively.

**Table 1:** Average classification performance for different classifiers.

**Classifier**	**Accuracy (%)**	**Precision (%)**	**Recall (%)**
LDA	65.9	68.1	58.4
SAE	69.2	73.6	58.8
MLP	64.9	67.8	56.2

SAE, when configured as described, outperformed both LDA and MLP on the testing dataset (McNemar statistical tests; *p* < 0.01).

## 4. Discussion

The aim of the experiments was to evaluate if stacked autoencoders perform better for the P300 detection than two other classifiers. Unlike common P300-based BCI systems, the classifiers were trained on a dataset merged from four subjects and subsequently tested on different 11 subjects without any further training. Therefore, it can be observed how the P300 detection system can perform when dealing with the data from previously unknown subjects. Most parameters for classification were manually adjusted during the time-consuming mainly empirically-driven process of trying different settings and observing results on the validation set.

As the results indicate, stacked autoencoders were consistently able to outperform multi-layer perceptrons. The improvement can be seen in both Figure 7 and Table 1. This difference can probably be explained by improved training in SAE that also includes unsupervised pre-training. The improvement is more pronounced in precision than in recall. Furthermore, SAEs were also able to outperform LDA. Tests revealed that both differences were statistically significant for the testing dataset used (*p* < 0.01). As Figure 7 illustrates, SAEs yielded higher accuracy than other classifiers in 9 out of total 11 subjects. Consequently, it appears that stacked autoencoders were able to match or outperform current state-of-the-art classifiers for the P300 detection in accuracy. These results are consistent with promising results reported by Sobhani (2014). For deep belief networks, the authors reported 60–90% for other methods compared with 69 and 97% for deep learning. The achieved results encourage using deep learning models for the P300 component detection with applications to P300-based BCIs.

Furthermore, during the process of manually adjusting parameters, it was observed that the comparative benefits of SAE increased with the increase in the dimensionality of feature vectors. This may be because linear classifiers such as LDA suffer from the curse of dimensionality (Ji and Ye, 2008). In contrast, SAE by itself also performs dimensionality reduction (Zamparo and Zhang, 2015).

Although classification accuracy is very important for the reliability of P300 BCI systems, only BCIs with reasonably fast bit-rate are comfortable to use for disabled users. Since real-world BCI systems should be able to evaluate ERP trials on-line, computational time for the processing and classification of feature vectors should not be higher than inter-stimulus intervals. According to our experience, to be comfortable to use, inter-stimulus intervals should be at least 200 ms. In the literature, only slightly lower inter-stimulus intervals are used for the P300 speller (for example, 175 ms in Sellers et al., 2006). Fortunately, once the BCI system is trained, classifying a single feature vector is usually not very time consuming. This is also relevant for feed-forward neural networks. Therefore, according to our experience, SAE, MLP and LDA can all be used in on-line BCI systems.

For the future work, more issues remain to be addressed. Although stacked autoencoders are less prone to overtraining than MLPs, during the fine-tuning phase, accuracy peaked after approximately 100 iterations and then leveled off slowly. Therefore, more regularization techniques for avoiding overfitting may be used. Despite being better than LDA and MLP, still, only four participants reached an accuracy above 70% which is often seen as a minimum to use a P300-based BCI (Lakey et al., 2011). It can therefore be evaluated how an individualized BCI system (i.e., the system trained on the data from the particular user) would perform and if better performance of SAEs outweighs their increased training times. In Sobhani (2014), pre-training possibilities for deep belief networks are discussed. The authors proposed that the weights of a new neural network could be initialized using the results of pre-training based on another subject. The same principle could be applied to stacked autoencoders. This could lead to possibly increased classification performance. Another possible strategy for increasing accuracy and bitrate would be to shorten the inter-stimulus interval. Although shorter intervals could lead to lower P300 amplitudes, SAE can classify high-dimensional feature vectors and could detect only slight differences in the feature vectors. Furthermore, it could also be interesting to explore stacked denoising autoencoders, deep belief networks or other deep learning training models. Finally, we plan to apply the presented methods to on-line BCI for both healthy and paralyzed subjects.

## Ethics statement

The manuscript uses only previously published datasets. In that case, no ethic committee was involved because the University of West Bohemia does not have an ethic committee. All participants signed the informed consent.

## Author contributions

LV and PM designed the experiment used to obtain the data. LV proposed, designed, and implemented the algorithm based on stacked autoencoders. LV wrote the manuscript. PM corrected the manuscript. Both authors read and agree with the final version of the manuscript.

### Conflict of interest statement

The authors declare that the research was conducted in the absence of any commercial or financial relationships that could be construed as a potential conflict of interest.
